# Evidences of a New Psychobiotic Formulation on Body Composition and Anxiety

**DOI:** 10.1155/2017/5650627

**Published:** 2017-09-24

**Authors:** Carmela Colica, Ennio Avolio, Patrizio Bollero, Renata Costa de Miranda, Simona Ferraro, Paola Sinibaldi Salimei, Antonino De Lorenzo, Laura Di Renzo

**Affiliations:** ^1^CNR, IBFM UOS of Germaneto, University “Magna Graecia” of Catanzaro, Campus “Salvatore Venuta”, Germaneto, 88100 Catanzaro, Italy; ^2^Comparative Neuroanatomy Laboratory of Ecology Department, University of Calabria, Ponte Pietro Bucci, Arcavacata di Rende, 87030 Cosenza, Italy; ^3^Department of Systems Medicine, University of Rome Tor Vergata, Via Montpellier 1, 00133 Rome, Italy; ^4^PhD School of Medical-Surgical Applied Sciences, University of Rome Tor Vergata, Via Montpellier 1, 00133 Rome, Italy; ^5^CAPES Scholarship (Proc No. BEX 13264/13-3), CAPES Foundation, Ministry of Education of Brazil, 70040-020 Brasília, DF, Brazil; ^6^Health Center Srl, Biomedical and Nutritional Center, 87100 Cosenza, Italy; ^7^Section of Clinical Nutrition and Nutrigenomic, Department of Biomedicine and Prevention, University of Rome Tor Vergata, Via Montpellier 1, 00133 Rome, Italy

## Abstract

**Background:**

Gut microbiota is implied in obesity, because of its ability to harvest energy from diet, and in the regulation of behavior. Given the link between gut microbiota, body composition, obesity, and anxiety, the aim of this study was to evaluate the effects of a new psychobiotic formulation.

**Methods:**

Eligible patients were randomly divided into three groups: psychobiotics oral suspension group (POSG); dietary treatment group (DTG); combined treatment group (CTG). All subjects underwent body composition and psychological profile evaluation.

**Results:**

Significant changes in body composition parameters in each group were relieved after all treatments. Hamilton anxiety rating scale (HAM-A) highlighted a significant reduction of the total score for all study population after treatments in POSG (*p* = 0.01) and CTG (*p* = 0.04). A reduction of HAM-A total score in anxious subjects in POSG or CTG and a significant reduction of positive subjects for HAM-A in POSG (*p* = 0.03) and in CDG (*p* = 0.01) were shown.

**Discussion:**

Three-week intake of selected POS represents a good approach to solve problems related to obesity and behavior disorders. However, new clinical trials need to be performed on a larger population and for a longer period of treatment before definitive conclusions can be made. This trial is registered with NCT01890070.

## 1. Introduction

Gut microbiota is an ensemble of 100 trillion microorganisms present in the gastrointestinal tract (GI), which belong to more than 1000 species and 700 strains [1], and plays a crucial role in human's physiology, due to its ability to maintain energy homeostasis and stimulate immunity as an endocrine organ, in a symbiotic relationship with the host [2]. Several factors like genetics, diet, infection, drug consumption, age, and sex could influence the nature of gut microbiota, both temporarily and definitely. An external change of its composition could induce a dramatic variation on the hosts' health [3, 4].

A growing body of evidence demonstrated the possible involvement of gut microbiota in fat mass accumulation and cardiometabolic disease onset. The World Health Organization described obesity as a disorder with an excessive body fat accumulation, abandoning the old definition, which restricted this condition to the simple body mass index (BMI) classification, to embrace a broader range of phenotypes, including subjects within the normal BMI range but with a critical percentage of body fat mass [5–10]. Obesity condition seems to be mostly caused by the obesogenic environment [11], which consists of a series of bad lifestyle habits from the disproportionate intake of calories, especially from simple carbohydrates, and the reduction of physical activity. Moreover, obese subjects could present some behavioral disorders [12], and some genetic profiles seem to be associated with the body weight regulation [13–16].

Gut microbiota seems to be implicated in obesity onset given its ability to harvest energy from the diet, through its influence on gut epithelium and motility [17], and increase triglyceride storage in the host adipocytes, inhibiting the fasting-induced adipose factors [18]. Furthermore, gut microbiota influences several metabolic processes such as lipogenesis, fatty acid oxidation, triglycerides, and cholesterol production [19]. Numerous *in vivo* studies observed that microbiota plays a strong role in adipose tissue accumulation. In fact, germ-free mice, although they ingest more calories than their littermates, result leaner than conventional mice. At the same time, microbiota transplantation from conventional to germ-free mice highlights a dramatic increase in body fat, triglyceride production, and insulin resistance, without changing their food habits [17, 20, 21].

In recent years, researchers focused their attention on the relationship between gut microbiota and brain development and function, discovering a bidirectional communication pathway between them, defined as microbiota-gut-brain axis.

Several studies highlighted the role of microbiota in the regulation of mood and behavior, like stress, anxiety, depression, and autism [22–24], as well as the potential therapeutic effects deriving from its modulation.

Due to the psychotropic effects in animal models and human clinical trials, the term “psychobiotics” was introduced [25]. In fact, it has been demonstrated that treatment with probiotics formulation, containing *Lactobacillus acidophilus*, *Lactobacillus casei*, and *Bifidobacterium bifidum*, showed positive effects on psychological distress [26, 27].

*In vivo* studies observed a relation between altered gut microbiota composition and anxiety related behavior, with increased exploration of aversive zones and improved serotoninergic function in germ-free mice when compared to specific pathogen-free counterparts [24, 28, 29].

Moreover, the anxiety-like behavior increases during pathogen infection and GI inflammation in animal models [30].

At the same time, anxiety and other psychological disorders seem to be related to body composition and obesity [31]. Several studies observed learning, memory, and function deficits in obese subjects, linking obesity to the exacerbation of depression and anxiety disorders [32–35]. Vice versa, depression disorders have a strong positive association with eating behaviors and fat mass, especially in subjects who do not follow a Mediterranean-like eating pattern [36].

Up today, very few studies demonstrated the beneficial effects of psychobiotics on the health status of obese subjects. An improvement of psychosocial behavior was seen in subjects with a fat mass surplus that underwent weight loss dietary treatments [37].

Given the link among gut microbiota, body composition, obesity, and the risk of developing anxiety, the aim of this study was to evaluate the differences deriving from the combination of 3-week administration of a new psychobiotic formulation, (psychobiotics oral suspension, POS) with or without dietary treatment (DT), consisting of a hypocaloric diet, on general population.

The evaluation was performed based on anthropometric, bioimpedance analysis (BIA), dual X-ray absorptiometry (DXA) measurements, and anxiety assessment with Hamilton anxiety rating scale (HAM-A).

## 2. Methods

### 2.1. Study Design and Subjects

This research was conducted using a prospective intervention study design, between January 2017 and April 2017. Forty-five subjects were recruited sequentially within a routine medical check-up program at the Section of Clinical Nutrition and Nutrigenomics, Department of Biomedicine and Prevention of the University of Rome “Tor Vergata.” POS was administrated 1 time/day, 2 h before lunch in order to ensure gastrointestinal transit and absorption.

Eligible patients were randomly divided into three groups (1 : 1 : 1 ratio): (1) psychobiotics oral suspension group (POSG), subjects took daily note 1 bag of 3 g of POS, and they did not change their ordinary diet; (2) dietary treatment group (DTG), subjects followed a hypocaloric diet; (3) combined treatment group (CTG), subjects followed the hypocaloric diet and took daily note 1 bag of 3 g of POS. Each group followed the assigned treatment consecutively for 3 weeks. At the beginning and at the end of each treatment, body composition evaluation and psychodiagnostic tests were performed.

Subjects were asked to maintain their usual lifestyle habits and to report any illness or abnormality arisen during the study.

The primary outcome of this study was the evaluation of nutritional status according to body composition changes measured by anthropometry, BIA, and DXA, due to the different treatments. The secondary outcome was the evaluation of anxiety disorder through the HAM-A test, pre- and posttreatment each.

All participants recruited in the study authorized their participation by reading and signing the informed consent, drafted in accordance with the provisions of the Ethics Committee of Medicine, University of Rome Tor Vergata and with the Helsinki Declaration of 1975 as revised in 1983. This trial is registered with NCT01890070, http://www.ClinicalTrials.gov.

### 2.2. Exclusion Criteria

Exclusion criteria included age < 20 y or >75 y, pregnancy, breastfeeding, type 1 diabetes, presence of intestinal bacterial overgrowth, characterized by high levels of hydrogen and methane production in the small bowel, acute diseases, endocrine disorders, liver, heart or kidney dysfunctions, history of chronic medication, antibiotic therapy up to ten days before enrollment, smoke, drug or alcohol abuse, and participation in another diet trial. No subjects with known alterations of intestinal transit following organic pathologies (abdominal surgery, diabetes mellitus, scleroderma, hypothyroidism, etc.) were included in the study. Subjects were advised not to consume any antibiotics or probiotics for the month prior to study initiation and to avoid using it for all the duration of the study.

### 2.3. Psychobiotics Oral Suspension Composition

1 bag of POS of 3 g contained: 1.5 × 10^10^ colony-forming unit CFU of *Streptococcus thermophilus* (CNCM strain number I-1630), 1.5 × 10^10^ colony-forming unit CFU of *Lactobacillus bulgaricus* (CNCM strain numbers I-1632 and I-1519); 1.5 × 10^10^ colony-forming unit CFU of *Lactococcus lactis* subsp. *lactis* (CNCM strain number I-1631); 1.5 × 10^10^ colony-forming unit CFU of *Lactobacillus acidophilus*; 1.5 × 10^10^ colony-forming unit CFU of *Streptococcus thermophiles*; 1.5 × 10^10^ colony-forming unit CFU of *Lactobacillus plantarum*; 1.5 × 10^10^ colony-forming unit CFU of *Bifidobacterium lactis* (CNCM I-2494); 1.5 × 1 10^10^ colony-forming unit CFU of *Lactobacillus reuteri* (DSM 17938), maltodextrin from corn, anticaking agent (silica), casein, lactose, and gluten < 3 ppm LLOQ (lower limit of quantitation), (Biocult strong, HOMEOSYN, Rome, Italy).

### 2.4. Anthropometric Analysis

According to the International Society for the Advancement of Kinanthropometry protocol and National Institute of Health Guidelines, waist circumference (WC) and hip circumference (HC) were taken using a flexible steel metric tape to the nearest 0.5 cm. Body weight (Kg) was measured to the nearest 0.1 Kg, using a technical balance (Invernizzi, Rome, Italy). Waist/hip ratio (WHR) was also evaluated in relation to clinical risk thresholds, that is, WHR > 1 for men and WHR > 0.9 for women. Height (m) was measured to the nearest 0.1 cm using a stadiometer (Invernizzi, Rome, Italy). BMI was calculated using the formula: BMI = body weight/height^2^ (Kg/m^2^).

### 2.5. Bioelectrical Impedance Analysis (BIA)

Resistance, reactance, impedance, phase angle, total body water (TBW), intracellular water (ICW), and extracellular water (ECW) were assessed by BIA phase sensitive system (BIA 101S, Akern/RJL Systems, Florence, Italy) [38, 39]. Impedance index (II) was evaluated with the following formula [40]:

(1) $$\begin{array}{c} II=\frac{{height}^{2}\;\left({cm}^{2}\right)}{resistance\;\left(\Omega \right)}. \end{array}$$

Measurements were taken according to Di Renzo et al. [39].

### 2.6. Dual X-Ray Absorptiometry (DXA)

Body composition analysis was assessed by DXA (i-DXA, GE Medical Systems, Milwaukee, WI, USA) according to the previously described procedures [12].

Total body fat (TBFat), total body lean (TBLean), android body fat (ABF), and gynoid body fat (GBF) were expressed as a percentage (%) of the total body mass. TBFat percentage was estimated by the ratio between the TBFat (Kg) and the sum of TBFat (Kg), TBLean (Kg) and bone mineral content (BMC) (Kg) multiplied by 100. The intermuscular adipose tissue (IMAT) was evaluated according to Bauer et al. [41].

### 2.7. Dietary Intervention

The energy intake for DT was calculated based on basal metabolic rate (BMR) of each subject, with Weir's formula: BMR = [(3.94 × VO2) + (1.106 × VCO2)] × 1.44 VO2, where VO2 is the volume of oxygen uptake (mL/min), estimated by the following formulas:

(2) $$\begin{array}{c} {VO}_{2}\;woman=TBLean\;DXA\times 4.5,\\ {VO}_{2}\;man=TBLean\;DXA\times 5.3, \end{array}$$

and VCO2 is the volume of carbon dioxide output (mL/min), evaluated with the following formula:

(3) $$\begin{array}{c} {VCO}_{2}={VO}_{2}\times 0.85. \end{array}$$

Protein intake was determined considering 2 g of protein/Kg of TBLean, representing 21–26% of daily caloric intake. Carbohydrate intake was between 44% and 51% of total energy intake, and fat intake was between 27% and 31% of daily caloric intake (<10% of saturated fatty acids, <300 mg/day of cholesterol). The fiber intake was 30 g/day [42].

### 2.8. Psychodiagnostic Instruments

Hamilton anxiety rating scale (HAM-A) was administered by instructed physicians and was used to measure the severity of anxiety symptoms. The 14 items on the scale define several symptoms and measure psychic and somatic anxiety. Each item has a score from 0 to 4, respectively, from the absence to the severe presence of the related symptom. The total score, which has a range from 0 to 56, describes three different scenarios: <17 indicates mild anxiety severity, 18–24 from mild to moderate anxiety severity, and 25–30 from moderate to high anxiety severity. In this work, we considered anxious subjects those who had a score higher than or equal to 18 [43, 44].

### 2.9. Statistical Analysis

Nonparametric tests for asymmetrically distributed data were conducted in all analyses and presented as median (minimum and maximum). Kruskal Wallis test was carried out to compare the three groups at baseline. To evaluate differences before and after 21 days of treatment, Wilcoxon test was performed in each group. To describe, quantitatively, variable change after treatments, we used a ratio of the absolute variation to the baseline value (percent variation = Δ%). Categorical variables were compared among groups by Chi-square (*χ*^2^) or Fisher's test. McNemar test was used for the comparison between groups at baseline (T0) and after treatment (T1). Statistical analyses were carried out using IBM SPSS 21.0 for Windows (Armonk, NY: IBM Corp. USA). In all statistical tests performed, the null hypothesis was rejected at the 0.05 level of probability.

## 3. Results

**Figure 1:**
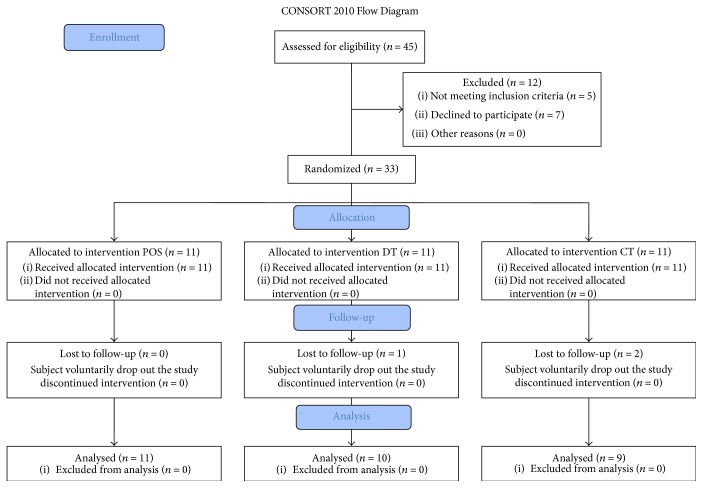
Study flow diagram according to Consort, 2010.

Out of forty-five subjects recruited, twelve were excluded from the trial: five did not meet inclusion criteria, seven declined to participate, and other three subjects voluntarily stopped the treatment (Figure 1).

During the trial, three subjects dropped out of the study and, finally, thirty patients between 21 and 72 years old with a BMI between 18.5 and 39.9 Kg/m^2^ and without metabolic complications met the inclusion criteria and completed the trial. No changes to trial outcomes occurred after it commenced. The median age of subjects was 45 years, 83.3% female and 16.7% male (Table 1). At baseline, no statistical significance was observed between groups (Table 2).

Significant changes in body composition parameters in each group were relieved after all treatments. In fact, POSG and CTG showed a significant reduction in II (*p* = 0.03 and *p* = 0.01, resp.), whereas both DTG and CTG highlighted a significant reduction in weight (*p* = 0.01), BMI (*p* = 0.01), waist circumference (*p* = 0.01 and *p* = 0.04, resp.), TBFat (Kg) (*p* = 0.03 and *p* = 0.04, resp.), and IMAT (*p* = 0.03; *p* = 0.04, resp.). Significant reduction in hip circumference (*p* = 0.02) and TBLean (Kg) (*p* = 0.02) were observed only in DTG, whereas waist/hip ratio (*p* = 0.04), PA (*p* = 0.02), ABFat (Kg) (*p* = 0.04), and GBFat (Kg) (*p* = 0.04) parameters were significantly reduced in CTG (Table 3).

**Figure 2:**
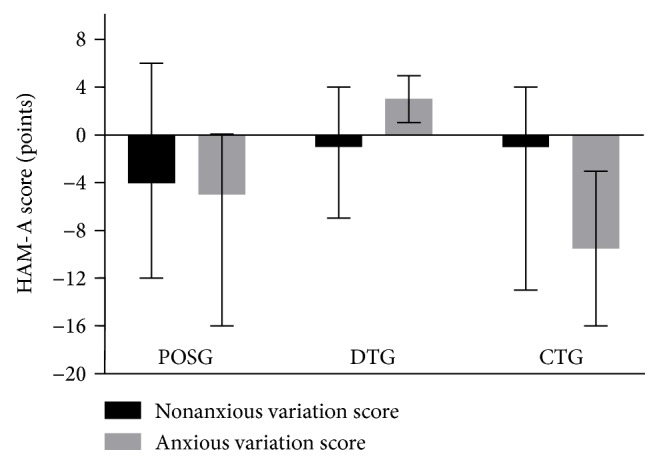
Hamilton anxiety rating scale (HAM-A) score variation before and after treatments in anxious and nonanxious subjects. Nonanxious subjects (negative test) if total score < 18 and anxious subjects (positive test) if total score ≥ 18. Variation score is shown as median, minimum, and maximum. 609 statistical significance attributed to results with *p* < 0.05 by Kruskal Wallis test. Anxious variation score among groups: *p* = 0.10 and nonanxious variation score among groups: *p* = 0.67. POSG: psychobiotics oral suspension group; DTG: dietary treatment group; CTG: combined treatment group.

The HAM-A test performed on POSG and CTG highlighted a significant reduction in the total score for all study population after both treatments (*p* = 0.01 and *p* = 0.04, resp.). No significant difference was seen in the HAM-A of DTG (Table 3). However, according to the total score, the sample was divided into anxious (total score ≥ 18) and nonanxious subjects (total score< 18) within each group. At baseline, no statistical significance was observed between POSG, DTG, and CTG for anxious and nonanxious subjects (*p* = 0.06). A notable reduction in the HAM-A total score in anxious subjects that had undergone the POS or combined treatment (Δ = −5 points and Δ = −9.5 points, resp.) was highlighted, while anxious DTG patients had HAM-A total score increased (Figure 2).

**Figure 3:**
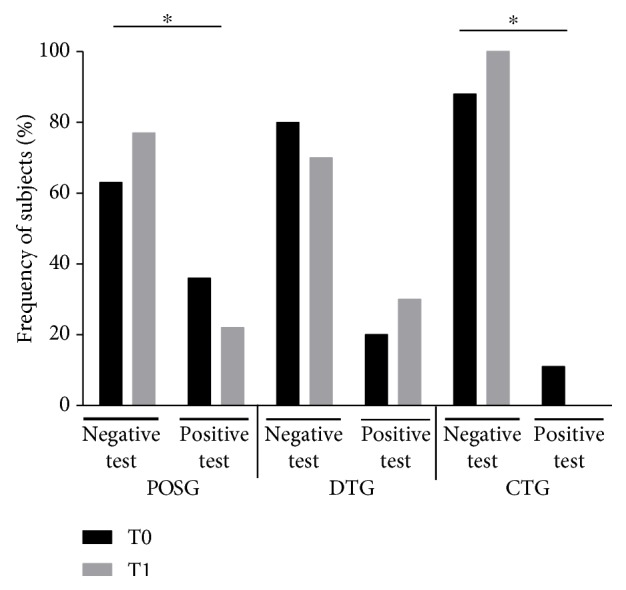
Frequency change of anxious subjects in POSG, DTG, and CTG after treatment. Frequency of anxiety was evaluated before and after treatment in POSG, DTG, and CTG. Negative test (nonanxious 619 subjects) if total score < 18 and positive test (anxious subjects) if total score ≥ 18. Statistical significance attributed to results with ^∗^*p* < 0.05 between T0 and T1 by McNemar test. POSG:*p* = 0.03^∗^; DTG: *p* = 0.10; CTG: *p* = 0.01^∗^. POSG: psychobiotics oral suspension group; DTG: dietary treatment group; CTG: combined treatment group.

Table 4 shows absolute numbers of anxious and nonanxious subjects in the 3 groups, before and after treatment. Furthermore, a significant reduction in the number of anxious subjects was observed in POSG (*p* = 0.03; Δ% = −39.3%), as well as in CTG, where all anxious subjects became nonanxious (*p* = 0.01; Δ% = −100%) (Figure 3).

## 4. Discussion

Neurotransmitters and neuromodulators, secreted by bacteria, are able to modulate the state of the hosts' mood: gamma-aminobutyric acid is produced by certain *Lactobacillus* and *Bifidobacterium* species; norepinephrine is released by *Escherichia*, *Bacillus*, and S*accharomyces* spp.; 5-hydroxytryptamine is released by *Candida*, *Streptococcus*, *Escherichia*, and *Enterococcus* spp.; and dopamine is produced by *Bacillus* and acetylcholine by *Lactobacillus* [45].

Fat mass increase in humans is related to several environmental factors, especially bad lifestyle habits, like sedentary living and excess of daily caloric, carbohydrate, and fat intake [11]. It is well known that the increase of body fat mass represents a strong risk factor for the development of metabolic and cardiovascular diseases [46–48].

Gut microbiota could play an essential role in fat mass increase and obesity development by invading the intestinal mucosa and causing systemic inflammation. On the other hand, the integrity of the intestinal barrier and a healthy intestinal microflora induce an anti-inflammatory effect that causes a consequent reduction in fat mass body composition [49].

Gut microbiota has been studied for decades in order to evaluate its impact on different aspects of human health and body composition [18–20], and recently, the role of probiotics with or without diet has been evaluated in terms of changing the overall health status [50, 51], body weight, body composition, and obesity [52, 53].

In our study, we enrolled normal weight, preobese, and obese up to the second-degree patients based on BMI, and, at the same time, we performed DXA to evaluate body composition. At baseline, the population resulted homogeneous in the three groups for the studied variables as reported in Table 2. On average, the population chosen had a TBFat percentage over 30%, who are considered obese according to De Lorenzo et al. [54].

The statistical comparison among the three groups exhibited that the subjects who belong to POSG did not report significant differences between time T0 and T1, in weight, waist and hip circumference, and body composition, except for the II (*p* = 0.03, Δ% = −1.92%). In accordance with the literature [55], the DTG demonstrated significant reductions in weight (*p* = 0.01; Δ% = −2.27%), BMI (*p* = 0.01; Δ% = −3.60%), waist circumference (*p* = 0.01; Δ% = −6.10%), hip circumference (*p* = 0.02, Δ% = −3.24%), TBFat (Kg) (*p* = 0.03, Δ% = −.32%), IMAT (*p* = 0.03; Δ% = −1.64%), and TBLean (Kg) (*p* = 0.02, Δ% = −1.64).

In the CTG, a higher variation of TBFat loss (*p* = 0.04, Δ% = −18.06%) and a statistically significant difference in the reduction of android (Δ% = −28.46%) and gynoid fat mass (Δ% = −11.46%) (*p* = 0.04) were highlighted. This preliminary data underlines the role of probiotics as a supplement for diet, as described by Kim et al. [56]. The 21-day period treatment is probably not enough to point out the positive effect of probiotic treatment alone on the improvement of weight and body composition, taking into account that most pharmacological treatments for obesity have a duration of at least three months, and in all cases, it is advised that they are coupled with lifestyle changes [57].

IMAT was significantly reduced in subjects treated only with diet (*p* = 0.03, Δ% = −1.64%), whereas the group under diet with probiotic intake showed a greater IMAT reduction (*p* = 0.04, Δ% = −21.77%). This result is the first evidence reported in the literature, and we speculate that it could be attributed to the capacity of probiotics to decrease the intestinal permeability with a consequent reduction of lipopolysaccharide and inflammatory cytokine levels [58].

The improvement of inflammatory state and oxidative status induced by probiotics administration [59, 60] could be able to contribute to the proper fatty acids and glucose metabolism, with the improvement of insulin resistance related to a better IMAT [61]. In CTG, the significant reduction of ABFat and waist/hip ratio, both related to insulin resistance and cardiovascular risk [62], could be due to an improvement of insulin profile.

BIA results show that POS intervention led to a significant reduction in II and, consequently, a resistance increase. The same trend was marked by Valentini Neto et al. [63], even if not significant.

However, a significant decrease of phase angle was observed in CTG, probably due to body water loss which translates into a resistance increase, despite of TBFat loss. Since in POSG we observed a phase angle reduction and a significant reduction of II, we can hypothesize a synergic action between diet and POS.

Multiple studies have demonstrated the existence of a clear link between gut microbiota and brain function. Given this, the gut microbiota appears to be a key regulator of mood and behavior [22]. Moreover, probiotics due to their effects on gut microbiota seem to have a positive impact on the management of psychological disorders, such as anxiety. In our study, POS supplementation led to a significant decrease of HAM-A score both in POSG than in CTG. This could be attributed to the effects of POS on molecular pathways in the central nervous system, which could also act on gut microbiota [23, 24]. We did not observe any significant difference in DTG on HAM-A score. This confirms that POS might have played a role in anxiety rather than only diet.

**Figure 4:**
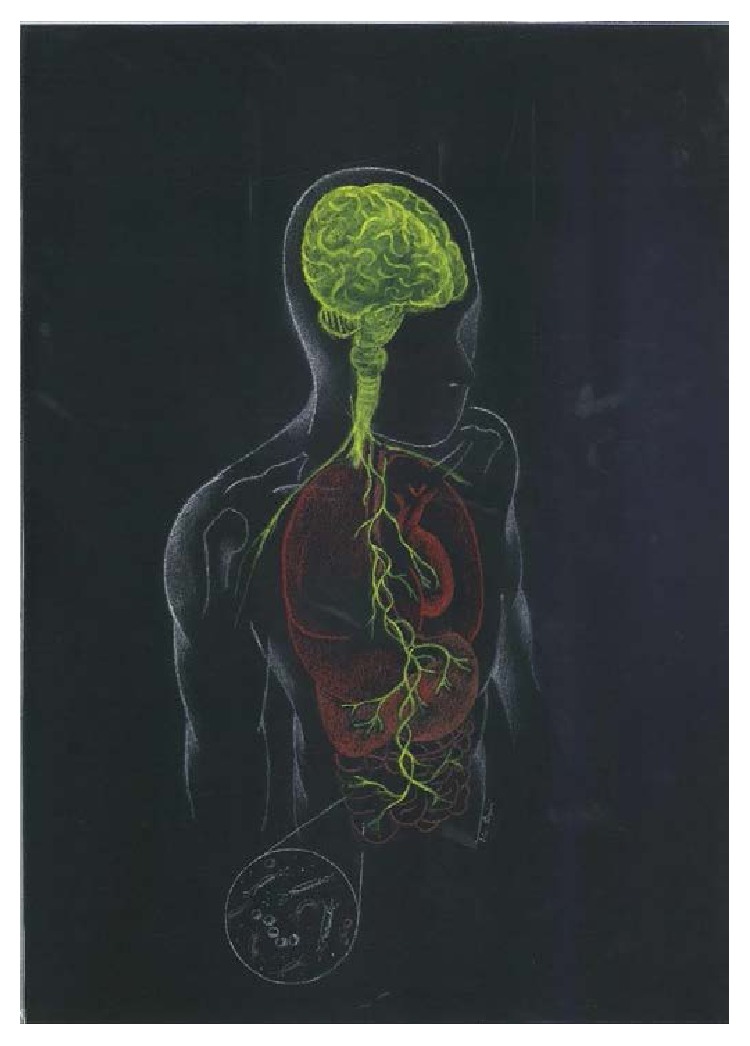
Gut-microbiota axis.

Based on the positive anxious score (total score ≥ 18), we observed that in both groups with POS, there was a significant decrease in the number of subjects that had a positive HAM-A test, with a score improvement of 5 points in POSG and 9.5 points in CTG. These results confirm that POS supplementation is associated with a reduction of anxiety, as shown by Wang et al. [64]. However, a balanced diet, associated with POS, seems to have a greater effect on the improvement of anxiety symptoms (Figure 4).

## 5. Conclusions

Despite the limitations of our study, related to the study design and the low sample size, our results highlighted that a three-week intake of selected psychobiotics represents a good approach to solve the problems related to obesity and behavior disorders. However, new clinical trials need to be performed on a larger population and for a longer period of treatment before definitive conclusions can be made.

## Figures and Tables

**Table 1:** Overall description of anthropometric, body composition, and anxiety data.

	Overall
	*n* = 30
	Median (min–max)
Age (years)	45.00 (21.00–72.00)
Weight (Kg)	77.75 (50.30–121.00)
Height (cm)	165.00 (150.00–186.00)
BMI (Kg/m^2^)	26.87 (20.12–39.93)
WC (cm)	89.25 (69.50–122.50)
HC (cm)	108.50 (85.50–132.00)
WHR	0.82 (0.69–1.09)
Phase angle (°)	7.05 (5.10–930)
II (cm^2^/Ω)	53.97 (39.35–94.28)
TBFat (Kg)	28.45 (14.03–44.64)
ABFat (Kg)	2.50 (0.70–4.38)
GBFat (Kg)	5.20 (2.72–7.70)
IMAT	1.21 (0.46–2.04)
TBLean (Kg)	43.23 (35.63–58.13)
HAM-A (points)	11.00 (0.00–30.00)

All results were expressed as median (minimum–maximum). BMI: body mass index; WC: waist circumference; HC: hip circumference; WHR: waist-to-hip ratio; II: impedance index; TBFat: total body fat; ABFat: android body fat; GBFat: gynoid body fat; IMAT: intermuscular adipose tissue; TBLean: total body lean; HAM-A: Hamilton anxiety rating scale (negative test (nonanxious subjects) if total score < 18 and positive test (anxious subjects) if total score ≥ 18).

**Table 2:** Anthropometric, body composition, and anxiety analysis of the 3 groups.

	POSG	DTG	CTG	
	*n* = 11	*n* = 10	*n* = 9	*p*
	Median (min–max)	Median (min–max)	Median (min–max)	
Age (years)	42.00 (26.00–65.00)	36.50 (21.00–72.00)	51.00 (44.00–72.00)	0.12
Weight (Kg)	67.80 (50.30–111.80)	77.00 (62.20–99.00)	79.50 (57.20–121.00)	0.45
Height (cm)	166.00 (155.00–186.00)	166.50 (150.00–173.00)	165.00 (161.00–185.00)	0.81
BMI (Kg/m^2^)	25.11 (20.12–39.45)	28.60 (26.10–38.60)	29.20 (22.07–39.93)	0.10
WC (cm)	77.00 (69.50–121.00)	94.25 (85.00–106.50)	90.00 (74.00–122.50)	0.27
HC (cm)	104.50 (85.50–132.00)	108.00 (102.00–120.00)	112.25 (89.00–132.00)	0.27
WHR	0.77 (0.69–1.05)	0.86 (0.80–0.94)	0.82 (0.71–1.09)	0.25
Phase Angle (°)	6.50 (4.60–10.20)	7.05 (5.10–9.30)	6.90Z (4.90–9.00)	0.71
II (cm^2^/Ω)	61.54 (39.35–69.05)	57.12 (42.79–82.53)	52.76 (45.40–94.28)	0.91
TBFat (Kg)	23.29 (14.03–24.57)	29.52 (23.00–44.64)	34.39 (15.05–44.19)	0.13
ABFat (Kg)	1.36 (0.70–2.40)	2.60 (1.29–4.23)	2.53 (0.82–4.38)	0.17
GBFat (Kg)	3.90 (2.97–4.95)	5.70 (4.35–7.70)	5.86 (2.72–7.05)	0.25
IMAT	0.89 (0.46–2.00)	1.22 (0.87–1.79)	1.47 (0.50–2.04)	0.25
TBLean (Kg)	35.88 (34.43–51.36)	43.23 (35.63–58.13)	41.58 (39.59–54.96)	0.35
HAM-A (points)	11.00 (3.00–28.00)	13.50 (7.00–30.00)	6.00 (00.00–23.00)	0.06

All parameters were evaluated at baseline among the 3 groups, by Kruskal Wallis test. All results were expressed as median (minimum–maximum). Statistical significance attributed to results with ^∗^*p* < 0.05. POSG: psychobiotics oral suspension group; DTG: dietary treatment group; CTG: combined treatment group; BMI: body mass index; WC: waist circumference; HC: hip circumference; WHR: waist-to-hip ratio; II: impedance index; TBFat: total body fat; ABFat: android body fat; GBFat: gynoid body fat; IMAT: intermuscular adipose tissue; TBLean: total body lean; HAM-A: Hamilton anxiety rating scale (negative test (nonanxious subjects) if total score < 18 and positive test (anxious subjects) if total score ≥ 18).

**Table 3:** Comparison between before and after treatment in each group.

	POSG (*n* = 11)			DTG (*n* = 10)			CTG (*n* = 9)		
	T0	T1	*p*	T0	T1	*p*	T0	T1	*p*
	Median (min–max)	Median (min–max)		Median (min–max)	Median (min–max)		Median (min–max)	Median (min–max)	
Weight (Kg)	67.80 (50.30–111.80)	66.30 (49.70–107.90)	0.39	77.00 (62.20–99.00)	75.25 (60.50–93.30)	0.01^∗^	79.50 (57.20–121.00)	76.50 (56.10–119.50)	0.01^∗^
BMI (Kg/m^2^)	25.11 (20.12–39.45)	24.75 (20.12–38.52)	0.39	28.60 (26.10–38.60)	27.57 (23.90–35.80)	0.01^∗^	29.20 (22.07–39.93)	28.10 (21.64–38.58)	0.01^∗^
WC (cm)	77.00 (69.50–121.00)	78.00 (68.00–121.00)	0.07	94.25 (85.00–106.50)	88.50 (80.00–100.00)	0.01^∗^	90.00 (74.00–122.50)	86.50 (71.00–115.00)	0.04^∗^
HC (cm)	104.50 (85.50–132.00)	105.00 (87.00–121.50)	0.40	108.00 (102.00–120.00)	104.50 (96.00–116.00)	0.02^∗^	112.25 (89.00–132.00)	109.50 (88.00–119.00)	0.17
WHR	0.77 (0.69–1.05)	0.76 (0.66–1.05)	0.28	0.86 (0.80–0.94)	0.84 (0.76–0.93)	0.09	0.82 (0.71–1.09)	0.81 (0.74–1.00)	0.04^∗^
Phase angle (°)	6.50 (4.60–10.20)	6.30 (4.90–8.60)	0.15	7.05 (5.10–9.30)	6.50 (4.70–8.60)	0.06	6.90 (4.90–9.00)	5.70 (5.10–6.60)	0.02^∗^
II (cm2/Ω)	51.52 (39.35–69.05)	50.53 (39.35–66.92)	0.03^∗^	57.12 (42.79–82.53)	53.43 (40.99–76.08)	0.06	55.11 (45.40–94.28)	51.93 (43.42–91.02)	0.01^∗^
TBFat (Kg)	23.29 (14.03–24.57)	22.70 (14.43–25.50)	0.59	29.52 (23.00–44.64)	29.13 (22.76–40.33)	0.03^∗^	34.39 (15.05–44.19)	28.18 (13.60–43.05)	0.04^∗^
ABFat (Kg)	1.36 (0.70–2.40)	1.24 (0.67–2.33)	0.11	2.60 (1.29–4.23)	2.65Z (1.23–3.60)	0.07	2.53 (0.82–4.38)	1.81 (0.61–4.23)	0.04^∗^
GBFat (Kg)	3.90 (2.97–4.95)	3.91 (2.90–4.87)	0.28	5.70 (4.35–7.70)	4.90 (4.10–7.77)	0.07	5.86 (2.72–7.05)	5.20 (2.43–6.76)	0.04^∗^
IMAT	0.89 (0.46–2.00)	0.85 (0.47–1.18)	0.59	1.22 (0.87–1.79)	1.20 (0.86–1.69)	0.03^∗^	1.47 (0.50–2.04)	1.15 (0.44–1.72)	0.04^∗^
TBLean (Kg)	35.88 (34.43–51.36)	35.87 (35.39–50.63)	1.00	43.23 (35.63–58.13)	42.61 (35.62–57.21)	0.02^∗^	41.58 (39.59–54.96)	40.42 (39.51–54.11)	0.22
HAM-A	11.00 (3.00–28.00)	7.00 (1.00–28.00)	0.01^∗^	13.50 (7.00–30.00)	10.00 (6.00–31.00)	0.72	6.00 (00.00–23.00)	5.00 (0.00–19.00)	0.04^∗^

All parameters were evaluated before and after treatments by Wilcoxon test. All results were expressed as median (minimum–maximum). Statistical significance attributed to results with ^∗^*p* < 0.05 between T0 and T1. POSG: psychobiotics oral suspension group; DTG: dietary treatment group; CTG: combined treatment group; BMI: body mass index; WC: waist circumference; HC: hip circumference; WHR: waist-to-hip ratio; II: impedance index; TBFat: total body fat; ABFat: android body fat; GBFat: gynoid body fat; IMAT: intermuscular adipose tissue; TBLean: total body lean; Ham-A: Hamilton anxiety rating scale (negative test (nonanxious subjects) if total score < 18 and positive test (anxious subjects) if total score ≥ 18).

**Table 4:** Absolute numbers of anxious and nonanxious subjects in all groups, before and after treatment.

	POSG		DTG		CTG	
HAM-A score	*n* = 11		*n* = 10		*n* = 9	
	T0	T1	T0	T1	T0	T1
HAM-A < 18	7	9	8	7	8	9
HAM-A ≥ 18	4	2	2	3	1	0

Frequency of anxiety subjects was evaluated before and after treatment in POSG, DGT, and CTG. Negative test (nonanxious subjects) if total score < 18 and positive test (anxious subjects) if total score ≥ 18. HAM-A: Hamilton anxiety rating scale; POSG: psychobiotics oral suspension group; DTG: dietary treatment group; CTG: combined treatment group.

## Abbreviations

BMI: Body mass index
POS: Probiotics oral suspension
DT: Dietary treatment
BIA: Bioimpedance analysis
DXA: Dual X-ray absorptiometry
HAM-A: Hamilton anxiety rating scale
POSG: Psychobiotics oral suspension group
DTG: Dietary treatment group
CTG: Combined treatment group
WC: Waist circumference
HC: Hip circumference
WHR: Waist-to-hip ratio
II: Impedance index
TBFat: Total body fat
TBLean: Total body lean
ABF: Android body fat
GBF: Gynoid body fat
BMC: Bone mineral content
IMAT: Intermuscular adipose tissue
BMR: Basal metabolic rate.
